# The effect of yogurt fortified with *Lactobacillus acidophilus* and *Bifidobacterium sp*. probiotic in patients with lactose intolerance

**DOI:** 10.1002/fsn3.2145

**Published:** 2021-01-20

**Authors:** Seyed Jalil Masoumi, Davood Mehrabani, Mehdi Saberifiroozi, Mohammad Reza Fattahi, Fariba Moradi, Masoud Najafi

**Affiliations:** ^1^ Nutrition Research Center School of Nutrition and Food Sciences Shiraz University of Medical Science Shiraz Iran; ^2^ Gastroenterohepatology Research Center Shiraz University of Medical Sciences Shiraz Iran; ^3^ Stem Cell Technology Research Center Shiraz University of Medical Sciences Shiraz Iran; ^4^ Burn and Wound Healing Research Center Shiraz University of Medical Sciences Shiraz Iran; ^5^ Comparative and Experimental Medicine Center Shiraz University of Medical Sciences Shiraz Iran; ^6^ Digestive Disease Research Center Digestive Disease Research Institute Tehran University of Medical Science Tehran Iran; ^7^ Office of Vice President for Health Affairs Shiraz University of Medical Sciences Shiraz Iran; ^8^ Student Research Committee Shiraz University of Medical Sciences Shiraz Iran

**Keywords:** bifidobacterium, *lactobacillus acidophilus*, lactose intolerance, probiotic

## Abstract

This study assessed the effect of probiotic yogurt fortified with *Lactobacillus acidophilus* and *Bifidobacterium sp*. in patients with lactose intolerance. Fifty‐five patients suffering from lactose intolerance were randomly divided into control group of 28 lactose intolerance patients who received nonprobiotic yogurt (100 ml) and experimental group consisted of 27 lactose intolerance patients who received probiotic yogurt fortified (100 ml) with *L. acidophilus* and *Bifidobacterium sp*. Each individual received yogurt for one week. Lactose intolerance was confirmed when the patients received 75 g lactose and were positive after 30 min until 3 hr for lactose intolerance symptoms and by hydrogen breath test (HBT). After intervention, the hydrogen level was lower in experimental group in comparison with the control group. Lactose intolerance symptoms in experimental group were much less than the control group. Our findings revealed that probiotic yogurt fortified with *L. acidophilus* and *Bifidobacterium sp*. could safely and effectively decrease lactose intolerance symptoms and HBT, so our probiotic can be recommended as a treatment of choice in lactose intolerance patients.

## Graphical abstract

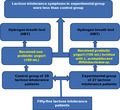

## 1 INTRODUCTION

Lactose is a disaccharide (glucose and galactose) sugar found in several foodstuffs and its absorption is dependent on the proper activity of the lactase enzyme in the small intestine. The lactase hydrolyses the lactose facilitating the passage of the carbohydrate through the intestine to blood circulation. The activity of lactase in mammals is at its peak during breast‐feeding, and fall after weaning resulting to a reduction in the capacity to lactose absorption (Delacour et al., 2017). This problem takes place genetically in humans and is known as primary hypolactasia or lactase nonpersistence (LNP) (Delacour et al., 2017).

The nonabsorbed lactose in the small intestine passes to the colon, where it is metabolized by the intestinal flora producing short‐chain fatty acids and gas, primarily hydrogen (H2), carbon dioxide (CO2) and methane (CH4), which are responsible for the symptoms of lactose intolerance that is an inherited autosomal recessive trait with incomplete penetrance (Case llas et al., 2009). Lactose intolerance is still common worldwide, and the primary form is the most common one (Case llas et al., 2009). The genetically programmed reduction in lactase activity during adulthood affects 75% of the world adult population and can cause severe digestive disorders, which are the sign of lactose intolerance and intolerance (Case llas et al., 2009).

Lactose intolerance symptoms vary depending on the residual lactase activity, the small bowel transit time, amount of ingested lactose, processing of lactose in colon (Wiley, 2020), and fermentation of lactose in the colon by its microbiota (Gingold‐Belfer et al., 2020). In suspected lactose‐intolerant subjects, lactose breath hydrogen test has been used as a very simple and useful method of diagnosis (Amini et al., 2019). Modulating the composition and metabolism of the colonic microbiota may affect lactose intolerance (Chen et al., 1999), and modulating colonic microbiota may be done through the targeted use of dietary supplement including probiotics (Culligan et al., 2009).

Various treatment modalities were reported for lactose intolerance including lactase supplementation, low‐lactose diet, and potentially, colonic adaptation by probiotics (Misselwitz et al., 2019). According to the FAO/WHO definition, probiotics are live microorganisms if adequately administered, they can confer a health benefit on the host (Culligan et al., 2009). Probiotics are bacteria, molds, or yeasts that are considered as live microbial food supplements or their components while lactic acid bacteria are the most common one consumed in fermented milk, yogurt, or other fermented food stuffs (Davani‐Davari et al., 2019). In probiotic preparations, the most common organisms were reported as Lactobacillus, Escherichia, Bifidobacterium, Bacillus, Enterococcus, Streptococcus, and some fungal Saccharomyces strains (Arvez et al., 2006), while Lactobacillus and Bifidobacterium are commensal bacteria in gut flora playing key roles in human health (Imani Fooladi et al., 2011).

Cano‐Contreras et al. included 48 patients, while 33 received the probiotic and 15 the placebo and demonstrated the probiotic to be efficacious and safe to decrease lactose intolerance symptoms in patients with lactose intolerance, but did not change the hydrogen breath test (HBT) (Cano‐Contreras et al., 2020). In a systematic reviews and meta‐analyses, an overall positive correlation was confirmed between lactose intolerance and probiotics regarding specific strains and concentrations (Leis et al., 2020). The different uses of probiotics in gastrointestinal disorders, such as lactose intolerance, were previously shown, but data from these clinical trials have been controversial that needs to be clarified (Guarner et al., 2012).

The mechanisms of action of these probiotics are not still clear, but may modify the pH of intestine, expressing b‐galactosidase, having positive effects on intestinal activity and microbiota of colon (Levri et al., 2005). Data from numerous researchers revealed that some strains of lactic acid bacteria in fermented milk products can relieve lactose intolerance symptoms through secretion of bacterial lactase into the digestive system (Sen, 2019). As the available data and findings on the relationship between probiotic supplementation and clinical outcomes in lactose intolerance individuals are inconclusive, this study was conducted to evaluate the therapeutic effect of probiotic yoghurt fortified with *Lactobacillus acidophilus* and *Bifidobacterium sp*. in lactose intolerance patients.

## 2 MATERIALS AND METHODS

### 2.1 Study enrollment

From September 2017 to March 2018 in a randomized double‐blind clinical trial using table of random numbers, 55 patients with lactose intolerance who referred to Mottahari Clinic or Gastroenterohepatology Research Center of Shiraz University of Medical Sciences, Shiraz, Iran, were enrolled in a convenient sampling method. The exclusion criteria were age less than 18 and more than 60 years old, any chronic diseases such as renal or heart failure, immunodeficiency or cancers, history of antibiotic use from one month ago, concurrent use of H_2_‐blockers, phenytoin, warfarin or theophylline and history of gastric or intestinal surgeries. Guidelines of the Declaration of Helsinki were followed in the research. The study was approved in Ethics Committee of Shiraz University of Medical Sciences (SUMS.AC.IR.2013–178), and an informed written consent was provided from each participant.

### 2.2 Grouping and interventions

The patients were divided into two groups of control (28 lactose‐intolerant patients) who received nonprobiotic yogurt just fortified with *Lactobasilus bulgaricus* and *Streptococcus thermophiles* (100 ml) for one week in three packages per day and the experimental group (27 lactose‐intolerant patients) who received probiotic yogurt fortified with *L. acidophilus* and *Bifidobacterium sp*. (100 ml) for one week in three packages per day, while *Lactobasilus bulgaricus* and *Streptococcus thermophiles* were present too. The demographic information of all patients was recorded. Symptoms like diarrhea, flatulence, abdominal pain, nausea, and vomiting were recorded in a subjective way as 1 (lack of symptom), 2 (slight), 3 (moderate), and 4 (severe) symptoms. Both groups were evaluated for clinical symptoms and Hydrogen breath test (HBT) results. All lactose intolerance‐related symptoms were assessed daily and weekly.

HBT was used at referral to confirm lactose intolerance for all patients as a simple and available test in our clinic as described previously by feeding patients with 75 g lactose and further follow‐up for each 30 min until 3 hr (Arola, 1994). Only those with positive HBT were enrolled in this study. The HBT was performed weekly during a three weeks follow‐up, and any change in hydrogen level of HBT was assessed using the following formulas in two groups: $D\mathrm{ifference}={\mathrm{HBT}}_{\mathrm{after}}‐{\mathrm{HBT}}_{\mathrm{before}.}$ $\mathrm{Change}\left(\%\right)=\frac{\mathrm{Difference}}{{\mathrm{HBT}}_{\mathrm{before}}}\times 100.$

### 2.3 Preparation of probiotic yoghurt

Probiotic fortified yogurt was provided from Fars Pegah Dairy Company. Regarding the probiotic yogurt, the range of *L. acidophilus* count on the first day of production was 1.4 × 10^7^ –4.1 × 10^7^ that reached to 3.1 × 10^6^ – 4.2 × 10^6^ on its expiration date (after 14 days). These figures for *Bifidobacterium sp*. were 1.1 × 10^7^ – 1.6 × 10^7^ and 1.2 × 10^6^ – 1.9 × 10^6^, respectively. Regarding the control regular yogurt, the range of *L. bulgaricus* count on the first day of production was 3.5 × 10^6^ – 8.2 × 10^7^ that reached to 5.2 × 10^6^ – 5.2 × 10^6^ on its expiration date (after 14 days). These figures for *S. thermophiles* were 1.6 × 10^7^ – 2.6 × 10^8^ and 3.6 × 10^7^ – 5.9 × 10^8^, respectively (Table 1).

**TABLE 1:** The process of probiotic yogurt production

No	Date	Probiotic yogurt*				Regular yogurt			
		1 day after production		14 days after production (Expire date)		1 day after production		14 days after production (Expire date)	
		Lactobasilus acidophilus	Bifidobacterium sp.	Lactobasilus acidophilus	Bifidobacterium sp.	Lactobasilus bulgaricus	Streptococcus thermophilus	Lactobasilus bulgaricus	Streptococcus thermophilus
1	2017.10.07	1.9 × 10^7^	1.4 × 10^7^	3.9 × 10^6^	1.4 × 10^6^	1.4 × 10^7^	1.6 × 10^8^	3.4 × 10^7^	4.8 × 10^8^
2	2017.10.13	1.6 × 10^7^	1.2 × 10^7^	3.1 × 10^6^	1.8 × 10^6^	8.3 × 10^6^	2.5 × 10^7^	9.5 × 10^6^	3.6 × 10^7^
3	2017.10.22	1.4 × 10^7^	1.1 × 10^7^	3.5 × 10^6^	1.6 × 10^6^	2.3 × 10^7^	2.3 × 10^8^	4.5 × 10^7^	5.5 × 10^8^
4	2017.11.04	3.3 × 10^7^	1.3 × 10^7^	3.9 × 10^6^	1.3 × 10^6^	2.1 × 10^7^	2.6 × 10^8^	5.3 × 10^7^	4.3 × 10^8^
5	2017.11.12	4.1 × 10^7^	1.5 × 10^7^	4.2 × 10^6^	1.9 × 10^6^	3.5 × 10^6^	1.9 × 10^7^	5.2 × 10^6^	5.6 × 10^7^
6	2017.11.29	1.5 × 10^7^	1.3 × 10^7^	3.5 × 10^6^	1.5 × 10^6^	3.4 × 10^7^	2.5 × 10^8^	6.4 × 10^7^	4.7 × 10^8^
7	2017.12.09	2.9 × 10^7^	1.4 × 10^7^	3.1 × 10^6^	1.2 × 10^6^	4.6 × 10^6^	1.6 × 10^7^	7.2 × 10^6^	3.8 × 10^7^
8	2010.12.18	3.2 × 10^7^	1.6 × 10^7^	3.4 × 10^6^	1.7 × 10^6^	5.9 × 10^7^	2.2 × 10^8^	8.4 × 10^7^	5.9 × 10^8^
9	2017.12.24	1.5 × 10^7^	1.1 × 10^7^	3.2 × 10^6^	1.8 × 10^6^	8.9 × 10^6^	2.5 × 10^7^	9.1 × 10^6^	4.6 × 10^7^
10	2018.01.01	2.3 × 10^7^	1.3 × 10^7^	3.3 × 10^6^	1.6 × 10^6^	4.5 × 10^7^	1.7 × 10^8^	7.8 × 10^7^	3.9 × 10^8^
11	2018.01.08	3.1 × 10^7^	1.5 × 10^7^	3.9 × 10^6^	1.3 × 10^6^	4.3 × 10^7^	2.6 × 10^8^	8.5 × 10^7^	4.9 × 10^8^
12	2018.01.27	1.8 × 10^7^	1.2 × 10^7^	3.2 × 10^6^	1.3 × 10^6^	3.9 × 10^7^	1.7 × 10^7^	8.4 × 10^7^	3.8 × 10^7^
13	2018.02.04	2.5 × 10^7^	1.6 × 10^7^	3.5 × 10^6^	1.2 × 10^6^	5.9 × 10^6^	1.3 × 10^8^	8.8 × 10^6^	4.5 × 10^8^
14	2018.02.25	2.4 × 10^7^	1.6 × 10^7^	3.3 × 10^6^	1.5 × 10^6^	8.2 × 10^7^	2.4 × 10^8^	9.4 × 10^7^	5.2 × 10^8^

* Probiotic yogurt in addition to *Lactobasilus bulgaricus* and *Streptococcus thermophiles* contained *Lactobasilus acidophilus* and *Bifidobacterium sp*.

The probiotic bacteria were *L. acidophilus* and *Bifidobacterium sp*. which were commercially confirmed by laboratories of Fars Pegah Dairy Company. The procedure described by Gilliland and Speck was used to confirm the identity of the culture (Gilliland & Speck, 1977). After pasteurization of milk in 90°C, it was allowed to cool down to 42°C. In this temperature, the traditional starter or probiotic was added to the milk tank. All products were collected in 100 g packages and kept in 42°C until the pH reached to the acidity of choice (pH = 5.4). Then, they were transferred to a 4–6°C cold room and kept there for 12 hr. When the quality control was approved, they were transferred to the clinic in a cool chain to be administered for the subjects. In the clinic, a refrigerator was available to keep the yogurts before administration.

### 2.4 Statistical analysis

The data were analyzed using SPSS software (Version 23, Chicago, IL, USA). Normality was checked by Shapiro–Wilk test, if sample size was less than 25 individuals and Kolmogorov–Smirnov test in the case of more than 25 subjects. Chi‐square test was used to evaluate the relationship between qualitative data. Independent sample *t* test was used to compare the quantitative data. *p* value <0.05 was considered significant.

## 3 RESULTS

### 3.1 Grouping and interventions

**FIGURE 1:**
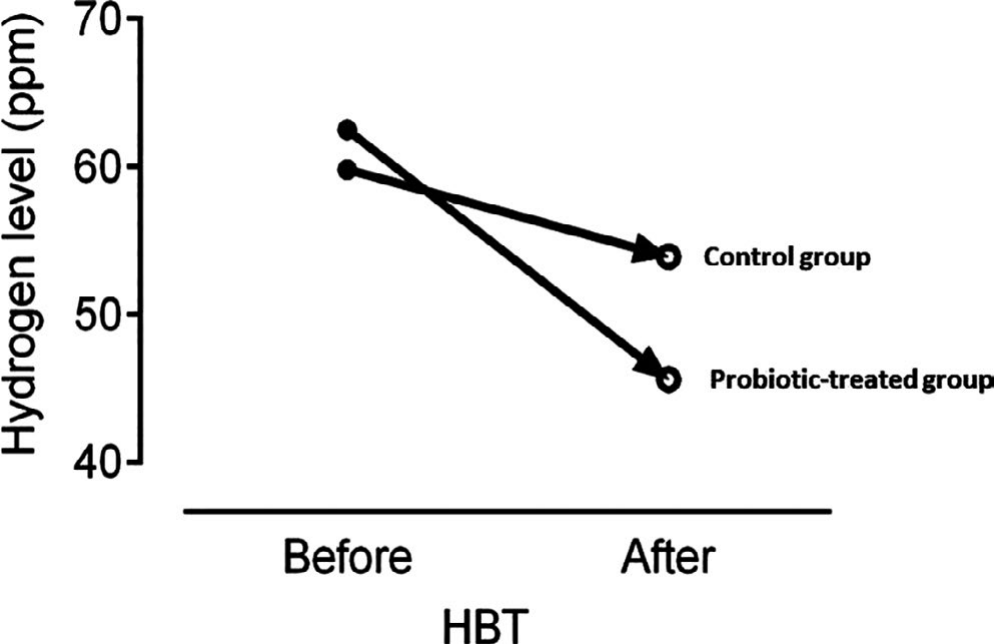
Comparison of hydrogen breath test (HBT) before and after consumption of yoghurt in the control (normal yoghurt) and probiotic‐treated group

**FIGURE 2:**
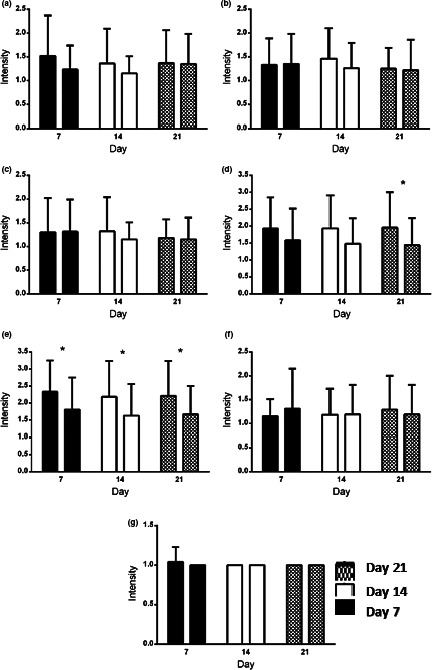
Comparison of mean and *SD* of gut symptoms between control and probiotic‐treated group after 1, 2, and 3 weeks. A: diarrhea; B: abdominal pain; C: cramps; D: bloating; E: flatulence; F: nausea; and G: vomiting. In each day, the left bar is related to the control group and the right bar is belonging to the probiotic‐treated group. The significant difference between two groups (*p* < .05) was indicated by asterisk

The demographic data of patients were presented in Table 2 denoting to absence of any significant difference between the 2 groups for age, gender, and history of previous diseases. As HBT findings in Figure 1 shows, on day 0 (before the intervention), the hydrogen level was 62.50 ± 43.52 ppm in experimental group and 59.82 ± 29.93 ppm in the control group (*p* = .67). One week after intervention, the hydrogen level in experimental group reached 45.61 ± 32.92 ppm and in the control group reached 53.93 ± 24.82 ppm (*p* = .31). Two weeks after intervention in the control group, the decrease in hydrogen level was not significant (*p* = .29), but this decline was significant in the experimental group (*p* = .02). Three weeks after intervention in the control group, the decrease in hydrogen level was not still significant (*p* = .21), but this decline was significant in the experimental group (*p* = .04). The mean and *SD* of the intensity of the gastrointestinal symptoms 1, 2, and 3 weeks after intervention were presented in Figure 2 demonstrating that the intensity of the gastrointestinal symptoms in experimental group was much lower than the control group for all symptoms except for abdominal pain and cramps after 1 week and nausea after 1 and 2 weeks. However, the difference was just significant for bloating after 3 weeks (*p* = .04) and flatulence after 1, 2, and 3 weeks (*p* = .04, *p* = .04, and *p* = .03, respectively).

**TABLE 2:** Demographic information of patients who were included in this study

Variable	Control group	Probiotic‐treated group	*p* value
*Age* (mean ± *SD*)	42.5 ± 11.1	41.4 ± 14.2	.74
Gender [*n* (%)]			
Female	21 (75%)	18 (66.7%)	.49
Male	7 (25%)	9 (33.3%)	
History of diseases [*n* (%)]			
No diseases	24 (82.1%)	26 (96.3%)	.29
Diabetes mellitus	2 (7.1%)	0 (0.0%)	
Appendicitis	1 (3.6%)	0 (0.0%)	
Gall bladder problems	1 (3.6%)	0 (0.0%)	
Large intestinal resection	0 (0.0%)	1 (3.6%)	

## 4 DISCUSSION

Nowadays, health knowledge has increased to a greater extent among consumers of healthy dairy and nondairy probiotic products (Ugidos‐Rodríguez et al., 2018). Commercial probiotics can be prepared as a powder, granule, capsule, liquid, gel, paste, or sachet (Gupta & Garg, 2009). Current dosages of probiotics range from 10^8^ to 10^11^ colony forming units (CFU) per day (Ndagijimana et al., 2009). In our study, the beneficial effects of *L. acidophilus* and *Bifidobacterium sp*. probiotic on the lactose intolerance symptoms were noted. *Bifidobacterium sp*. and *Propionibacterium sp*. have been isolated from the gut and fermented dairy products and have been used as a probiotic for intestinal health and treatment of gastrointestinal disorders (van de Guchte et al., 2012).

Ingestion of probiotics such as *Lactobacillus spp*., *Bifidobacterium longum* or *Bifidobacterium animalis* that produce lactase in the gut have been widely studied. Probiotics such as lactase‐positive bacteria including Lactobacillus, Bifidobacterium, and Streptococcus can be added to pasteurized dairy products to increase digestibility of the lactose (Rolfe, 2000). Of several studies involving probiotic interventions in lactose intolerance patients, five studies mentioned use of *L. acidophilus* (Kim & Gilliland, 1983; Lin et al., 1991, 1998; Montes et al., 1995; Pakdaman et al., 2016), one included *B. animalis* (Roškara et al., 2017), and one applied *B. longum* (Vitellio et al., 2019), denoting to varying degrees of efficacy but with an overall positive correlation between probiotics and lactose intolerance based on the strain and the concentration.

A recent systematic review confirmed an overall positive effect for probiotics such as *Lactobacillus spp*., *B. longum* or *B. animalis* that confirms proper use of probiotic yoghurt fortified with *L. acidophilus* and *Bifidobacterium sp*. in lactose malabsorption of our population of study (Oak & Jha, 2019). The probable mechanisms for the beneficial effects of these bacteria include the fermentation of lactose and also replacing or potentiating the normal flora and production of more lactase (de Vrese et al., 2001). Lactose intolerance should not be treated primarily based on reducing the intolerance, but should be focused on improving gastrointestinal symptoms (Deng et al., 2015).

Therefore, an interest has been on use of probiotic microbiota in therapeutic approach of gut symptoms. Probiotic strains with lactase enzyme have been successfully used in combination to improve the lactose intolerance and intolerance symptoms (de Vrese et al., 2015) similar to our study using probiotic yoghurt fortified with *L. acidophilus* and *Bifidobacterium sp*. in lactose intolerance. Oral administration of a probiotic fortified with *L. casei Shirota* and *B*. breve *Yakult* for 4 weeks showed improvement of the gut symptoms and decrease in the hydrogen level by HBT in lactose‐intolerant patients (Almeida et al., 2012) that is in consistency with our finding using probiotic microbiota in therapeutic approach of intolerance gut symptoms in therapeutic approach of intolerance gut symptoms.

Identical to our results, the positive effect of probiotics used in combination has been illustrated in 37 lactose‐intolerant children with chronic abdominal pain (Ockeloen & Deckers‐Kocken, 2012). Four‐week consumption of probiotic fortified with Lactobacillus and Bifidobacterium was demonstrated to improve symptoms and decrease hydrogen production in lactose‐intolerant patients persisting for at least 3 months after suspension of probiotic consumption (Almeida et al., 2012). Lactobacillus when used alone was shown to be effective in improving gastrointestinal symptoms in 60 lactose intolerance patients evaluated by HBT (Ojetti et al., 2009).

Similarly, we showed the effectiveness of Lactobacillus in reduction of symptoms in lactose intolerance patients. Among lactose intolerance symptoms, those related to gas production in the gastrointestinal tract including bloating and flatulence, probiotic use was shown to significantly prevent lactose fermentation (Shaukat et al., 2010; de Vrese et al., 2015). Our findings are also in line with these reports demonstrating an improvement in lactose intolerance symptoms and positive changes in hydrogen level detected by HBT after probiotic consumption. There is inconsistency with *L. acidophilus* consumption in milk showing not to be effective in reduction of gut symptoms in patients with self‐reported lactose intolerance (Shaukat et al., 2010).

The difference may be due to variation in enrollment criteria, outcome reporting, and the composition and dosing of the probiotic. *B. subtilis* has been safely used as a probiotic and has been well tolerated in the clinical subjects without undesirable physiological effects (Lefevre et al., 2017). Different probiotics were used in milk revealing that *L. plantarum* was a good candidate for probiotic yogurt fermentation (Wu et al., 2017). It was suggested that the inclusion of microencapsulated bacteria by complex coacervation in yogurts could become an effective vehicle for successful delivery of probiotics to the gut, and hence contributing to the improvement of the gastrointestinal tract health, without altering the texture of the product (Bosnea et al., 2017).

Even no adverse effects have been reported on use of probiotics in lactose intolerance and have been effectively used in alleviation of lactose intolerance symptoms, but still more studies with more participants seem necessary because of great variability among individuals receiving the probiotics (Roškara et al., 2017). So, future studies to assess the benefits of probiotics in multispecies combinations and in combination with other medications to understand their effect on the gut microbiota are needed (Didari et al., 2015).

The consumption of probiotic yoghurt fortified with *L. acidophilus* and *Bifidobacterium sp*. in lactose intolerance represented an efficient therapeutic strategy to improve the most annoying symptoms related to lactose intolerance such as bloating and flatulence without any side effects. Also, a decreased hydrogen level assessed by HBT was noted after consumption of fortified probiotic yoghurt. Therefore, our findings can be added to the literature on decrease in lactose intolerance symptoms and hydrogen level in response to probiotic yogurt fortified with *L. acidophilus* and *Bifidobacterium sp*.
